# Complete Remission With Fourth-Line Immunotherapy After Chemotherapy Failure in a Lung Cancer Patient With Severe Autoimmune Disease: An Unexpected Turn in Oncologic Management

**DOI:** 10.7759/cureus.86116

**Published:** 2025-06-16

**Authors:** Daniel Herrero Rivera, María Soriano Segura, Javier Orlando Prato Varela, Yaroslav Brygadyr Medvid, José Antonio López Martín

**Affiliations:** 1 Medical Oncology, HLA Moncloa University Hospital/ATRYS Health, Madrid, ESP; 2 Medicine, European University of Madrid, Madrid, ESP; 3 Pathology, HLA Moncloa University Hospital, Madrid, ESP; 4 Precision Medicine, ATRYS Health, Madrid, ESP

**Keywords:** autoimmune disease, complete response, immune related adverse events, immunotherapy, lung cancer

## Abstract

The indication for nivolumab in patients with advanced non-small cell lung cancer (NSCLC) who have progressed to first-line platinum-based systemic therapy was one of the first indications for immunotherapy approved by regulatory agencies. However, it is generally the case that patients with autoimmune diseases (AIDs) are excluded from studies due to the risk of immune exacerbations and a higher rate of immune-related adverse effects. This deprives these patients of the potential benefits they could obtain from immunotherapy, especially in those cases with favorable biomarkers of a good response. In this study, we present a clinical case of a patient with rapidly progressive multiple sclerosis (MS) of years of evolution, who obtained an impressive response to immunotherapy as a last therapeutic option, remaining cancer-free to date.

## Introduction

Immunotherapy has significantly transformed cancer treatment, particularly in malignancies such as non-small cell lung cancer (NSCLC). However, its use in patients with autoimmune diseases (AIDs) presents considerable challenges due to the potential for exacerbating underlying immune dysfunction. Immune checkpoint inhibitors (ICIs), including anti-PD-1/PD-L1 antibodies, have demonstrated efficacy across a range of malignancies. Nevertheless, by enhancing immune activity, they may induce immune-related adverse events (irAEs), which can mimic or worsen pre-existing autoimmune conditions [1]. As a result, patients with AIDs have traditionally been excluded from clinical trials involving immunotherapy, limiting the available data regarding safety and efficacy in this subgroup [2].

Despite these concerns, emerging evidence suggests that immunotherapy may be feasible in selected patients with well-controlled AID. For instance, a meta-analysis of observational studies indicated that although such patients may experience a higher incidence of irAEs, most events were manageable and rarely required treatment discontinuation [3].

In the context of NSCLC, immunotherapy has provided substantial survival benefits. Nevertheless, the presence of an underlying AID introduces additional complexity. An observational study found that patients with NSCLC and pre-existing AIDs who received ICIs exhibited response rates comparable to those without AIDs, though they experienced a slightly increased rate of irAEs [4]. 

It is important to note that not all autoimmune conditions confer the same level of risk. Patients with inactive or well-controlled AIDs not requiring high-dose immunosuppression may be appropriate candidates for ICIs [5]. Conversely, those with active disease or requiring intensive immunosuppressive therapy should be approached with greater caution.

In this situation, multiple sclerosis (MS) presents a particular challenge. MS is a chronic autoimmune demyelinating disease of the central nervous system, primarily driven by autoreactive T lymphocytes that escape central and peripheral tolerance. The immunopathogenesis of MS involves an imbalance between pro-inflammatory and regulatory immune pathways, including dysregulation of immune checkpoints such as PD-1 and CTLA-4, which normally function to restrain autoreactive T cells. The inhibition of these checkpoints, central to the mechanism of ICIs, may inadvertently lower the threshold for autoimmune activation, potentially triggering disease flares in susceptible individuals. This theoretical concern is supported by animal models and case reports describing MS reactivation or onset following ICI therapy [6]. 

This report presents the case of a patient with long-standing, rapidly progressive MS, who achieved an unexpected complete response to immunotherapy following the diagnosis of metastatic pulmonary adenocarcinoma with high PD-L1 expression and progression through three prior lines of chemotherapy.

## Case presentation

A 71-year-old woman, wheelchair-bound due to a rapidly progressive primary progressive MS (PPMS) diagnosed five years earlier, was referred to oncology for evaluation of a left lower lobe pulmonary mass identified on CT scan performed in September 2022. Comorbidities included Parkinsonism and a history of femoral fracture requiring osteosynthesis.

Initial blood work revealed normocytic anemia (hemoglobin (Hb) 10.1 g/dL), lymphopenia (460/μL), and elevated lactate dehydrogenase (LDH) (767 U/L). Tumor markers showed elevated carcinoembryonic antigen (CEA) (6.18 ng/mL) and cytokeratin fragment (CYFRA) 21.1 (4.76 ng/mL).

**Figure 1:**
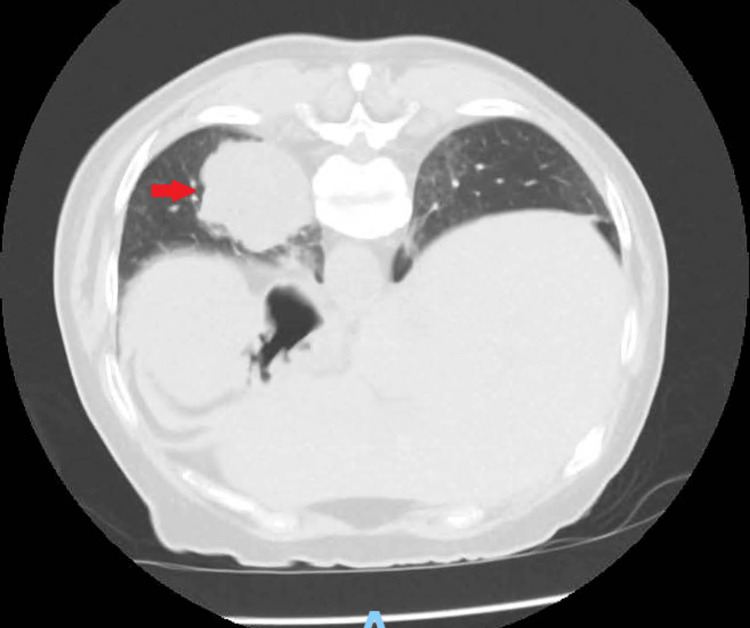
CT scan on September 27, 2022, showing mass of 5 x 4 cm in lower left lobe (red arrow) This image corresponds to the primary tumor before being operated on.

A CT scan on September 27, 2022 (Figure 1) and a positron emission tomography (PET) CT on September 29, 2022 showed a hypermetabolic pulmonary lesion of 5 x 4 cm (maximum standardized uptake value (SUV_max_) 11.7) with mediastinal nodal uptake (hilar SUV 6.8; subcarinal SUV 4). CT-guided biopsy yielded insufficient material for a definitive diagnosis but was suggestive of malignancy with TTF1 and cytokeratin positivity.

**Figure 2:**
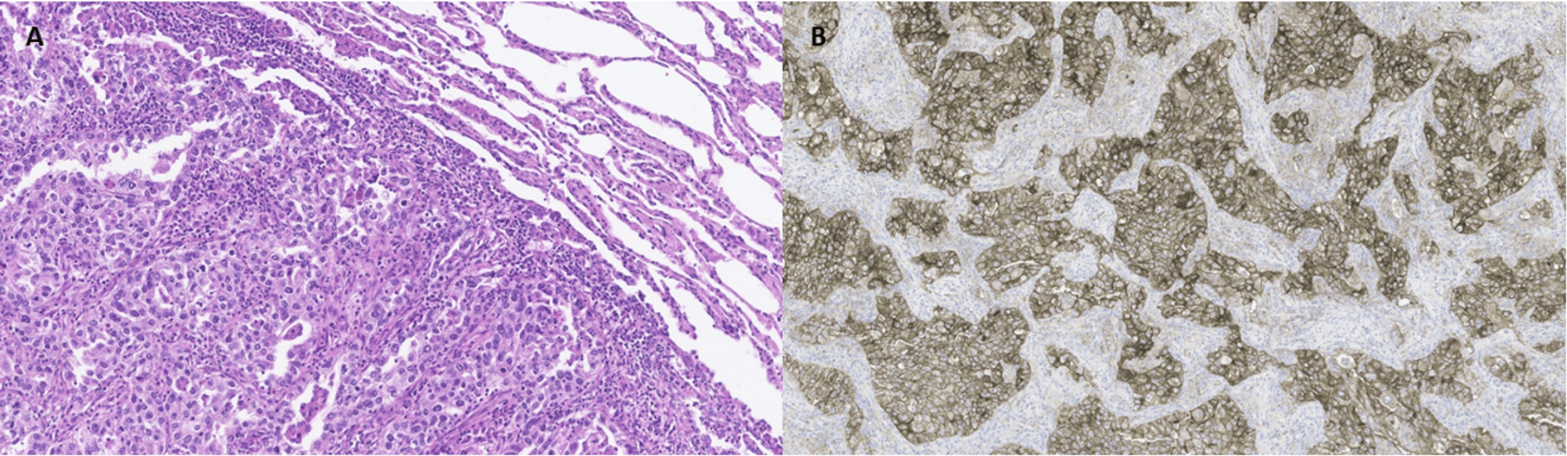
Histological section showing adenocarcinoma of solid and papillary pattern A) Microscopic (histological) examination: Histologically, the lesion was a neoplasm of epithelial origin, composed of atypical cuboidal or columnar cells with oval nuclei, irregular nuclear membranes, prominent nucleoli, and eosinophilic or clear cytoplasm. These cells were predominantly arranged in solid nests (solid pattern: 80%) or around fibrovascular cores (papillary/micropapillary pattern: 20%). The tumor showed involvement of the visceral pleura (PL2), with no clear evidence of lymphovascular invasion, perineural invasion, or STAS. No lymph nodes were submitted with the specimen. Immunohistochemical study: On immunohistochemical analysis, the tumor cells were positive for TTF-1 and napsin-A, and negative for p63, consistent with a diagnosis of primary lung adenocarcinoma. B) An additional immunohistochemical study for PD-L1 (PD-L1 22C3, DAKO) showed positivity in the majority of tumor cells (TPS: 90%). Final pathological diagnosis: High-grade (G3) pulmonary adenocarcinoma STAS: Spread through air spaces; TPS: Tumor proportion score

Despite inconclusive histology, high clinical suspicion of cT2bN2M0 lung cancer prompted referral to thoracic surgery. Considering that the patient was unable to properly undergo pulmonary function tests due to her comorbidity, and in the absence of a confirmatory histological oncological diagnosis, the thoracic surgery team performed an excisional lung biopsy through an atypical segmentectomy via video-assisted thoracoscopic surgery (VATS) on October 17, 2022. Pathology confirmed an 8 x 4.5 x 4.2 cm adenocarcinoma (Figure 2A), solid (80%) and papillary (20%) components, pleural invasion (PL2), without lymphadenectomy. Pathological stage was pT4 Nx M1a (pleural implant), R2 (due to unresected lymphadenopathies).

The concomitant use of adjuvant CTRT was ruled out due to the high toxicity and residual volume that requires very large fields.

Adjuvant chemotherapy with carboplatin and vinorelbine was initiated. Despite initial hematologic toxicity (thrombocytopenia and anemia), the patient completed four cycles. Given the high level of PD-L1 expression (tumor proportion score (TPS) 90%, (Figure 2B)), with EGFR and ALK wild type tested using next-generation sequencing (NGS) and the lack of effective alternatives, immunotherapy was considered as a treatment option after chemotherapy. However, since the resection was incomplete and she had a history of autoimmune neurological disease (PPMS), it was decided to postpone its administration until disease progression and the exhaustion of other therapeutic options. It was not possible to obtain definitive reports for the rest of the biomarkers analyzed by NGS due to initial problems with the health insurance company, but this could currently be remedied by claiming said results given that the patient's conditions have changed.

**Figure 3:**
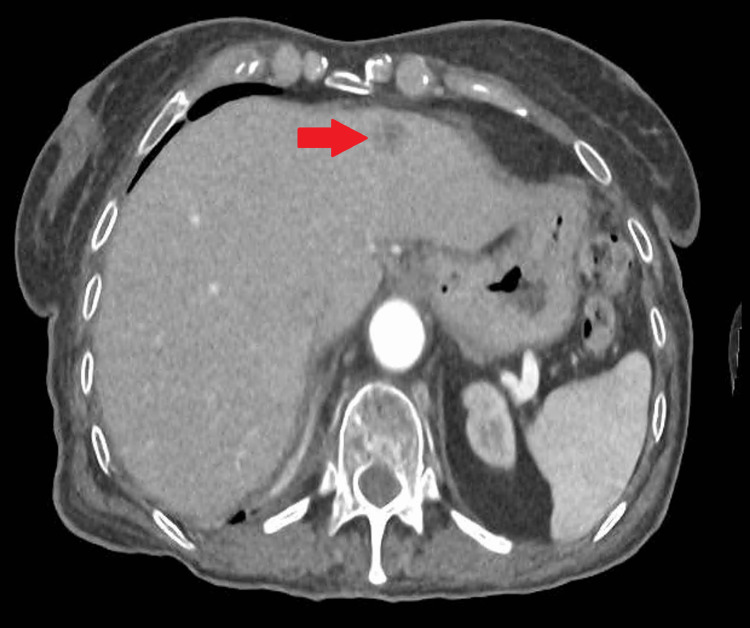
December 2023 CT scan showing disease progression with new hepatic lesions This image corresponds to the appearance of new metastatic liver lesions after receiving three cycles of pemetrexed. The red arrow corresponds to a new 13-mm metastatic lesion in segment III.

In June 2023, imaging showed progression with right adrenal 36 x 16 mm and retroperitoneal nodal metastases. She started second-line oral vinorelbine at a dose of 60 mg/m² on days one and eight of each 21-day cycle, receiving a total of five cycles, but progressed again by September 2023 to the retroperitoneal lymph node level. Third-line pemetrexed was initiated but halted after three cycles due to further progression in December 2023, including new hepatic of 13 mm in segment III and splenic metastases and increase of adrenal to 36 x 20 mm and retroperitoneal metastases (Figure 3).

**Figure 4:**
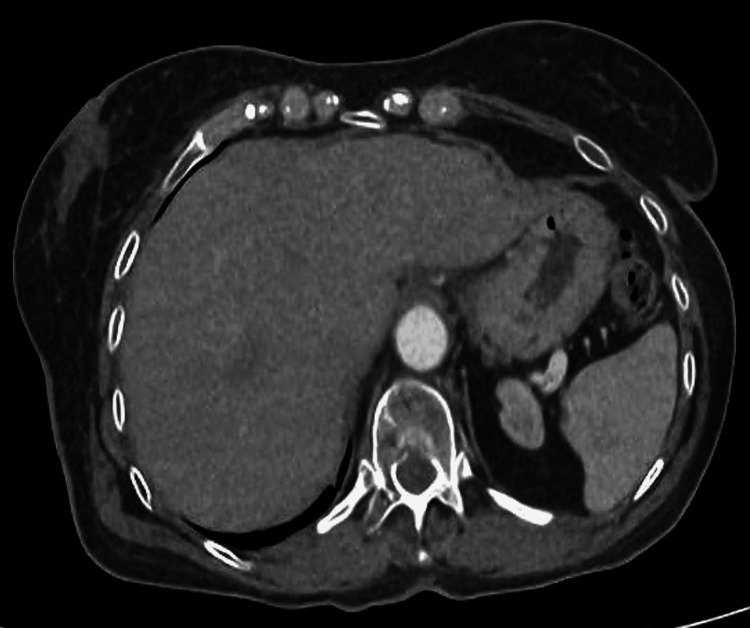
June 2024 CT scan showing complete response of hepatic lesions This image corresponds to the same plane as in Figure 3, with the disappearance of metastatic liver lesions after receiving 12 cycles of nivolumab.

Fourth-line nivolumab 240 mg every two weeks was started on December 26, 2023, after multidisciplinary consensus and neurologist approval. The patient experienced a grade two pruritus and mild cognitive impairment, which were manageable with antihistamines and supportive care. Imaging in February 2024 showed pseudoprogression with a small increase of hepatic subcapsular lesion but, in March and June 2024, showed a partial then complete radiological response, respectively, with disappearance of all metastatic lesions (Figure 4). The patient entered a therapeutic pause on August 16, 2024, after receiving 16 cycles of nivolumab over a period of 7.69 months.

Throughout treatment, her neurological condition remained stable. The last available evaluation in March 2025 confirmed continued disease remission.

## Discussion

ICIs have redefined the therapeutic landscape of advanced NSCLC, especially in tumors exhibiting high PD-L1 expression. However, patients with pre-existing AIDs have been consistently excluded from pivotal trials due to concerns about disease flares and elevated risk of irAEs [7].

MS, particularly in its progressive forms, presents a distinct challenge. Theoretical risks associated with PD-1 blockade include immune-mediated exacerbation, neurotoxicity, and paradoxical inflammatory responses. Nonetheless, retrospective data and case reports suggest that ICIs can be cautiously administered in patients with stable or inactive MS, particularly when oncologic alternatives are limited [8,9]. One of the largest case series reported in the literature to date of patients with MS treated with immunotherapy indicates that in the case of inactive AIDs, treatment tolerance is usually acceptable, although of the 12 cases described, only one achieved a complete response [10]. This makes our case rare given the few cases published in the literature that have responded in this way to immunotherapy and given the disadvantage of having received many previous lines of treatment. A review by Haanen et al. highlights that while 16-50% of patients with AIDs experience irAEs, most events are manageable and seldom require permanent discontinuation of therapy [11]. 

In this case, the decision to initiate nivolumab was based on multiple factors: a) absence of actionable mutations (EGFR/ALK wild-type); b) high PD-L1 expression (90%); and c) progression following three prior lines of chemotherapy. Moreover, the patient’s neurologist explicitly endorsed the use of ICIs in view of her neurologic stability. No MS relapses occurred during treatment, although mild neurocognitive symptoms and peripheral weakness were noted-likely attributable to disease progression and cervical spine degeneration rather than direct ICI toxicity.

Comparing our data with those from the Checkmate 057 clinical trial in patients with advanced non-squamous NSCLC that had progressed to platinum-based therapy, a good response to immunotherapy was expected, considering that in those with PD-L1 expression ≥50%, the objective response rate exceeded 40% and the hazard ratio (HR) for overall survival was 0.32 [12]. However, obtaining a complete response is what has drawn attention in our case, since only four patients (1.7%) included in this clinical trial achieved it, and in none of them after progression to three lines of prior systemic cytotoxic therapy. This indicates that if we estimate a good response based on biomarkers, we should consider administering immunotherapy regardless of any events that may have occurred previously.

A notable feature in this case was the occurrence of pseudoprogression, a phenomenon seen in approximately 5-10% of ICI-treated patients [13]. It is characterized by transient tumor enlargement or new lesion appearance, followed by regression. In this patient, newly identified hepatic lesions regressed completely without changing the treatment protocol, supporting the strategy of continuing immunotherapy beyond initial radiologic progression in clinically stable individuals [14].

Another issue to address in this case was what treatment to consider should our patient experience a new progression of her oncologic disease. One of the first options we would suggest is a rechallenge with immunotherapy, given the excellent response previously achieved and the acceptable tolerance shown by the patient. However, this therapeutic option lacks strong prospective clinical evidence [15]. There is a study conducted in advanced-stage NSCLC where retreatment with nivolumab in patients who had discontinued it after completing one year of therapy showed benefits, with up to a 36% survival rate at 13.5 months after rechallenge in a cohort of 39 patients [16]. Furthermore, European Society for Medical Oncology (ESMO) guidelines recommend that, in order to consider retreatment with immunotherapy, there should be at least a six-month treatment-free interval to optimize the chances of achieving a response [17]. Taking these premises into account, we would likely opt to attempt a rechallenge in our patient. 

Furthermore, this case illustrates the value of multidisciplinary care. Close collaboration among oncology, neurology, radiology, and rehabilitation teams facilitated comprehensive risk assessment, shared decision-making, and individualized supportive management. The patient's quality of life was preserved, and her functional status remained stable despite advanced disease and prolonged immunotherapy.

## Conclusions

Our case shows how, despite having a serious neurological AID, when we have no other alternatives other than immunotherapy in a patient with predictive biomarkers of good response, we should consider carrying out ICI treatment with very close monitoring, given that we could achieve good and long-lasting response rates, while maintaining acceptable tolerance.
